# Effectiveness of endovascular repair versus open surgery for the treatment of thoracoabdominal aneurysm: A systematic review and meta analysis

**DOI:** 10.1016/j.amsu.2022.104477

**Published:** 2022-09-03

**Authors:** Aayat Ellahi, Fahd Niaz Shaikh, Haider Kashif, Hamna Khan, Eman Ali, Bushra Nasim, Mariam Adil, Zunera Huda, Ayesha Liaquat, Muhammad Sameer Arshad

**Affiliations:** a Department of Medicine, Jinnah Sindh Medical University, Karachi, Pakistan; b Department of Medicine, Dow University of Health Sciences, Karachi, Pakistan

**Keywords:** Aneursym, Thoracoabdominal aneurysm, Endovascular repair, Open surgery

## Abstract

**Background:**

Thoracoabdominal aortic aneurysms (TAAAs) are associated with significant comorbidities. The aim of our study is to compare the outcomes of open repair versus endovascular repair of TAAAs.

**Methods:**

A thorough literature search was conducted on MEDLINE, Embase, and Cochrane Central databases. The analysis included observational studies comparing the outcomes of surgical vs endovascular aneurysm repair (EVAR) of TAAA. Mortality, spinal cord ischemia (SCI), renal failure, stroke, paraplegia, and respiratory and cardiac problems were all included in the studies. The results were provided as relative risks (RRs) with 95% confidence intervals (CIs). These were then aggregated using an inverse variance weighted random-effects model, and the pooled analysis was displayed using forest plots.

**Results:**

This meta-analysis compromising of twelve studies revealed significant results, favoring endovascular repair versus open surgery for all-cause mortality (HR = 1.91; 95% CI: 1.68–2.18; P < 0.00001), SCI (HR = 1.62; 95% CI: 1.18–2.21; P = 0.003), respiratory complications (HR = 2.22; 95% CI: 1.78–2.77; P < 0.00001), and cardiac complications (HR = 1.66; 95% CI: 1.38–2.00; P < 0.00001). Upon subgroup analysis based on propensity matched, results were consistent and significant for the outcomes of all-cause mortality, cardiac complications, and respiratory complications. For the propensity unmatched subgroup, the incidence of all-cause mortality, SCI, respiratory complications, and cardiac complications were lower among endovascular repair cohort.

**Conclusion:**

Current evidence supports the use of endovascular repair over open surgery. However, there is a need to conduct dedicated randomized controlled trials to effectively compare and determine the benefits and risk of both strategies.

## Highlights

• Current findings support endovascular repair over open surgery for the treatment of thoracoabdominal aneurysms (TAAA).
• Endovascular repair for TAAA can help reduce the risk of all-cause mortality, respiratory and cardiac complications.
• Consistent results were noted upon subgroup analyses.
• Dedicated randomized controlled trials are warranted to compare the benefits and risk of both strategies.

## 1 Introduction

Thoracoabdominal aortic aneurysm (TAAA) is a condition in which the descending thoracic aorta becomes dilated and abdominal aorta can also be involved [1]. TAAAs are rare events as globally only 5.9 cases are reported per 100,000 persons per year. However, more recent research show a rising prevalence of 10.4 occurrences per 100,000 in the United States [2]. Due to its complex anatomical location in the human body, it holds a significant risk of morbidity and mortality and demands urgent repair to prevent rupture of the aorta in people who have a degenerative aneurysm or a chronic aortic dissection [3]. Various techniques have been experimented with to find the best way possible to treat TAAAs and considering the technical challenges associated with post-operative care of patients in open surgery, improved short-term morbidity and reasonable durability have been achieved as a result of relatively recent development of endovascular and hybrid approaches [4].

Although, open surgical repair has been long considered as the preferred treatment strategy by physicians for TAAA due to its well-established durability [5] and a life expectancy >10 years [6] It has been linked to an increased risk of death and morbidities such as paraplegia and paraparesis of the spinal cord, new-onset renal failure necessitating dialysis, and stroke. Gastrointestinal ischemia is also another post-operative complication that can be lethal, despite its rarity [3]. Despite the benefits, data suggests that endovascular aortic repair has a higher risk of spinal cord impairment, and its durability remains uncertain. Even though endovascular methods offer some benefits over the more invasive surgical procedure, the short- and long-term benefits of the procedure are still unclear. A few studies have compared the post-operative results of both methods. Accordingly, we sought to examine the effect of surgical repair compared with the effect of endovascular repair of TAAA on pre- and post-operative outcomes.

## 2 Methods

This systematic review and meta-analysis has been reported in concordance with guidelines provided by the Preferred Reporting Items for Systematic Reviews and Meta-Analysis statement (PRISMA) [7] and the Risk of Bias in Systematic reviews and assessment of multiple systematic reviews (AMSTAR) 2 [8].

### 2.1 Data sources and search strategy

From the beginning of the course of this study through February 2022, MEDLINE, Embase, and Cochrane databases was searched independently by the two reviewers (HK and AE). No restrictions were set on time or language. MeSH terms and keywords were used in search strategy for thoracoabdominal, endovascular and aneurysm incorporated using Boolean operators. Other data sources were also scrutinized, namely bibliographies of editorials, conference proceedings of indexed abstracts, relevant reviews from major medical journals and databases of grey/unpublished/unprinted literature. This study was registered in National Institute for Health Research (NIHR) International prospective register of systematic reviews (PROSPERO) (Identification No. CRD42022341490) [9].

### 2.2 Study selection

The predefined inclusion criteria were: 1) observational studies published after January 2006; 2) patients with thoracoabdominal aneurysm who underwent either open or endovascular repair; 3) one of the outcomes mentioned later were included at least: mortality, spinal cord ischemia (SCI), renal failure, stroke, paraplegia, and respiratory and cardiac complications.

### 2.3 Data extraction and quality assessment

Two reviewers (HK and AE) underwent mass scrutinization, extracted and verified the data. To eliminate any event of discrepancies, the original articles were also reviewed. To calculate risk ratios with 95% confidence intervals (Cls), summary events and totals were extracted. Hazard ratios with 95% CIs were extracted and estimated to RRs, in the occurrences where summary events were not available. In addition, other study characteristics were also extracted (Table 1). The Newcastle- Ottawa scale was also used to evaluate the quality of this study based on pre-specified criterion of comparability, selection and outcome or exposure of included studies.

**Table 1:** Baseline characteristics.

**Reference**	**Year**	**Country**	No.		Age, years, mean +_SD		Female, No. (%)		COPD, No. (%)		DM, No. (%)	
			**Endo**	**Open**	**Endo**	**Open**	**Endo**	**Open**	**Endo**	**Open**	**Endo**	**Open**
**Observational matched**												
Ferrer [12]	2016	Italy	65	65	70.7+-7	70.7+-7	14 [22]	16 [25]	34(52)	30 (46)	5(8)	7(11)
Bertoglio [11]	2018	Italy	18	18	76 +-7	70+-4	4 [22]	3(17)	–	–	–	–
Rodolfo V Rocha [13]	2019	Canada	241	241	70.1 +-9.6	69.4 +-10.0	–	–	94 (39.0)	94 (39.0)	59 (24.5)	55 (22.8)
**Observational Unmatched**												
Ferrer [12]	2016	Italy	84	257	72.1+-14	66.2+-14	21 [25]	62 [24]	43(51)	127(49)	8 [10]	50(19)
Michel [21]	2015	France	268	1678	71.6+-9	69.2+-9	18 [7]	139(8)	61 [23]	241(14)	38(14)	210 [13]
Salata [22]	2012	Canada	20	11	69.1+-12	69.0+-14	8 (40)	1 [9]	–	–	3(15)	1 [9]
Sachs [23]	2010	United States	1167	3015	–	–	392 (34)	1046 (35)	249(21)	534(18)	124(11)	196(7)
Greenberg [24]	2008	United States	352	372	71.3+-12	62.7+-13	123 (35)	134 (36)	105 (30)	63(17)	39(11)	19(5)
Locham [25]	2017	United States	62	144	76.0+-13	72.0+-10	35 (57)	62 (43)	8(13)	28(19)	8(13)	9 [6]
Locham [26]	2018	United States	481	398	71.2+-10	66.5+-11	230 (48)	164 (41)	211 (44)	178(45)	76(16)	57(14)
Rodolfo V Rocha [13]	2019	Canada	303	361	71.3+-9.4	67.5 +-11.2	–	–	126 (41.6	138 (38.2)	80 (26.4	87 (24.1)
Dean J. Arnaoutakis [27]	2020		92	66	72+-8	59+-12	–	–	52 (57)	16 [24]	17 [18]	5 [8]
Geisbusch [6]	2019	Germany	839	1422	NK	NK	NK	NK	NK	NK	NK	NK
P. Chulhi Kang [28]	2019	United states	68	54	74.9 (68.2–78.5) (median,IQR)	62.0 (41.5–69.1) (median,IQR)	17 (25.0)	17.98 (33.3)	–	–	–	–

### 2.4 Statistical analysis

For Meta-analysis, RevMan (version 5.3; Copenhagen: Nordic Cochrane Centre, The Cochrane Collaboration) was used. The results were pooled using an inverse variance weighted random-effects model and given as RRs with 95% confidence intervals. Forest plots were used to visualize the combined analyses. The Higgins I2 method was employed to assess study heterogeneity. A score of 25–50% was considered low, 50–75% was considered moderate, and >75% was considered severe. To limit the possibility of bias, outcomes were separated into subgroups using propensity matched and unmatched data. For the SSE outcome, a funnel plot was used to measure publication bias. In all cases, a P-value of less than 0.05 was considered significant.

## 3 Results

### 3.1 Literature search results

**Fig. 1:**
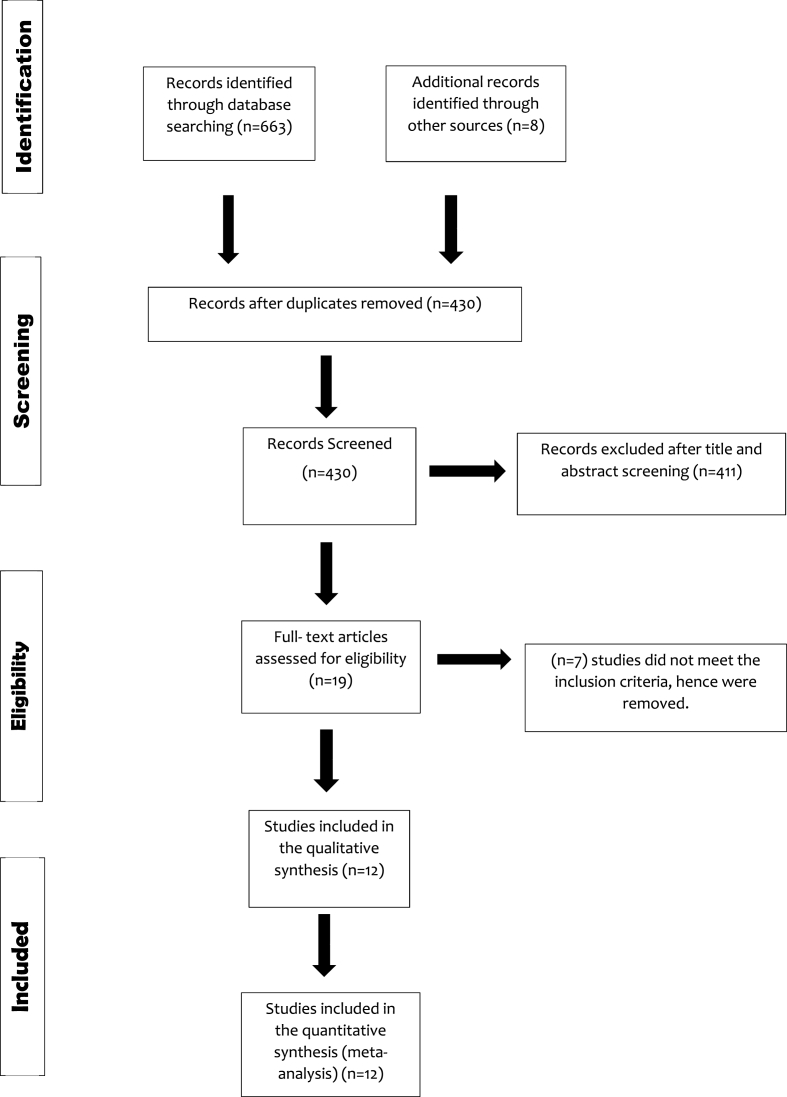
PRISMA flow Diagram.

The initial literature search turned in 671 publications that were relevant. 12 papers were chosen for inclusion in this meta-analysis after employing the established eligibility criteria. The PRISMA flow diagram depicts the entire literature search and study selection process. (Fig. 1)) (see .

### 3.2 Study characteristics and quality assessment

Table 1 summarizes the study's features and baseline demographics. Patients ranged in age from 69 to 74.9 years old. Observational studies were rated as being of moderate to high quality, with values ranging from 5 to 8 on the Newcastle Ottawa scale out of a maximum of 9 (Supplemental Table S1). As shown by funnel plot showed, Egger's regression was also non-significant for publication bias (p = t = 0.98; p = 0.58) (Supplemental Fig. S1). In all cases, a P-value of less than 0.05 was considered significant.

### 3.3 Results of meta-analysis

#### 3.3.1 All-cause mortality

**Fig. 2:**
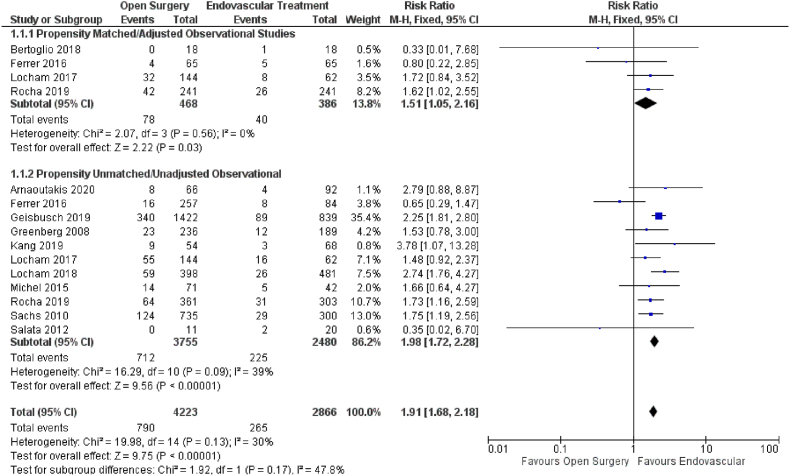
Forest plot comparing Open Surgery and Endovascular Treatment for All-cause Mortality.

Twelve studies reported the effect of open versus endovascular repair on all-cause mortality (Fig. 2). Endovascular repair in patients with TAAAs had a favorable significant impact on all-cause mortality (HR = 1.91; 95% CI: 1.68–2.18; P < 0.00001) when compared with open repair. Our results were consistent upon subgroup analysis based on propensity score matched (HR = 1.51; 95% CI: 1.05–2.16; P = 0.03) and unmatched data (HR = 1.98; 95% CI: 1.72–2.18; P < 0.00001).

#### 3.3.2 Spinal cord injury

**Fig. 3:**
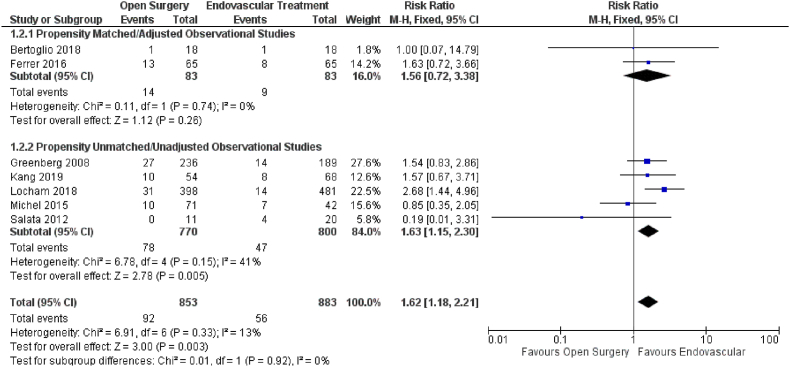
Forest plot comparing Open Surgery and Endovascular Treatment for Spinal Cord Injury.

The effect of open versus endovascular repair on the outcome of SCI was studied in seven studies (Fig. 3). When compared to open repair, endovascular repair had a significant positive influence on SCI (HR = 1.62; 95% CI: 1.18–2.21; P = 0.003) in patients with TAAAs. Subgroup analysis using propensity score unmatched data yielded similar outcomes (HR = 1.63; 95% CI: 1.15–2.30; P = 0.005). However, results differed for the subgroup analysis based on propensity score matching SCI (HR = 1.56; 95% CI: 0.72–3.38; P = 0.26).

#### 3.3.3 Renal failure

**Fig. 4:**
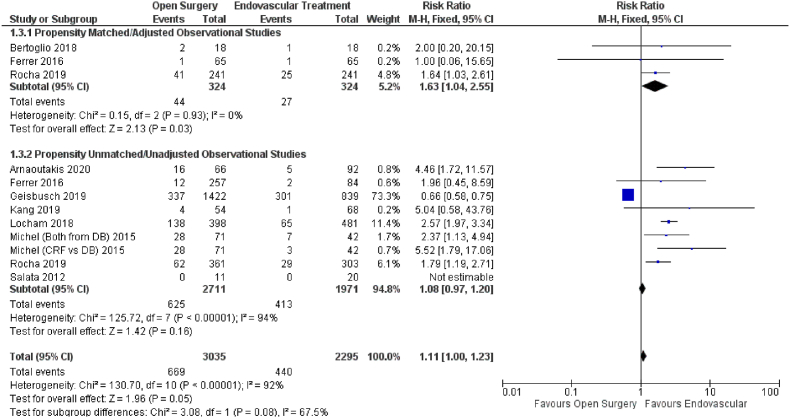
Forest plot comparing Open Surgery and Endovascular Treatment for Renal Failure.

The effect of open versus endovascular repair on the outcome of renal failure was studied in ten studies (Fig. 4). When compared to open repair, endovascular repair had no significant effect on renal failure in patients with TAAAs (HR = 1.11; 95% CI: 1.00–1.23; P = 0.05). Subgroup analysis using propensity score unmatched data yielded similar results (HR = 1.08; 95% CI: 0.97–1.20; P = 0.16). The subgroup analysis based on propensity score matching produced a significant outcome (HR = 1.63; 95% CI: 1.04–2.55; P = 0.03), preferring endovascular treatment over open repair.

#### 3.3.4 Stroke

**Fig. 5:**
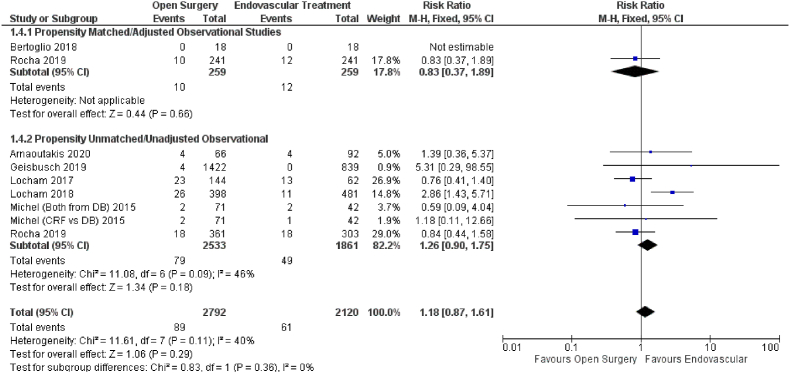
Forest plot comparing Open Surgery and Endovascular Treatment for Stroke.

Eight studies reported the effect of open versus endovascular repair on the outcome of stroke (Fig. 5). Endovascular repair in patients with TAAAs had no significant impact on stroke (HR = 1.18; 95% CI: 0.87–1.61; P = 0.29) when compared with open repair. Subgroup analysis using propensity score matched (HR = 0.83; 95% CI: 0.37–1.89; P = 0.66) and unmatched data (HR = 1.26; 95% CI: 0.90–1.75; P = 0.29) data yielded similar findings.

#### 3.3.5 Respiratory complications

**Fig. 6:**
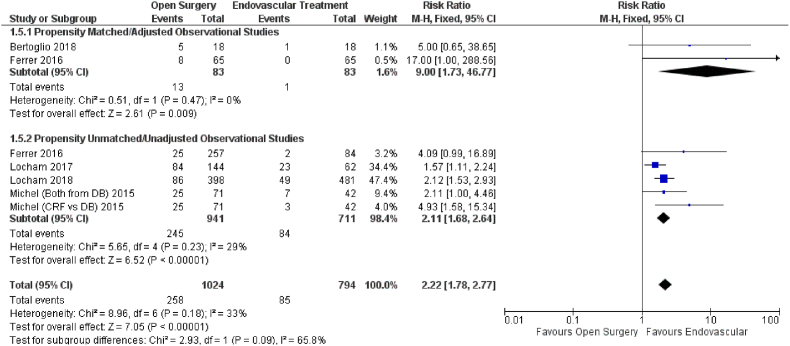
Forest plot comparing Open Surgery and Endovascular Treatment for Respiratory Complications.

The effect of open vs endovascular repair on stroke outcome was studied in eight studies (Fig. 6). When compared to open repair, endovascular repair had a significant influence on respiratory complications (HR = 2.22; 95% CI: 1.78–2.77; P < 0.00001) in patients with TAAAs. Subgroup analysis using propensity score matched (HR = 9.00; 95% CI: 1.73–46.77; P = 0.009) and unmatched data (HR = 2.11; 95% CI: 1.68–2.64; P < 0.00001) yielded similar results.

#### 3.3.6 Paraplegia

**Fig. 7:**
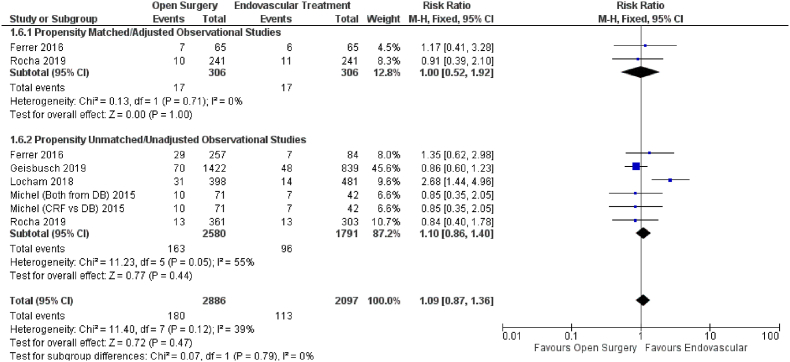
Forest plot comparing Open Surgery and Endovascular Treatment for Paraplegia.

Six studies reported the effect of open versus endovascular repair on the outcome of paraplegia (Fig. 7). Endovascular repair in patients with TAAAs had no significant impact on paraplegia (HR = 1.09; 95% CI: 0.87–1.36; P = 0.47) when compared with open repair. Our results were consistent upon subgroup analysis based on propensity score matched (HR = 1.00; 95% CI: 0.52–1.92; P = 1.00) and unmatched data (HR = 1.10; 95% CI: 0.86–1.40; P = 0.47).

#### 3.3.7 Cardiac complications

**Fig. 8:**
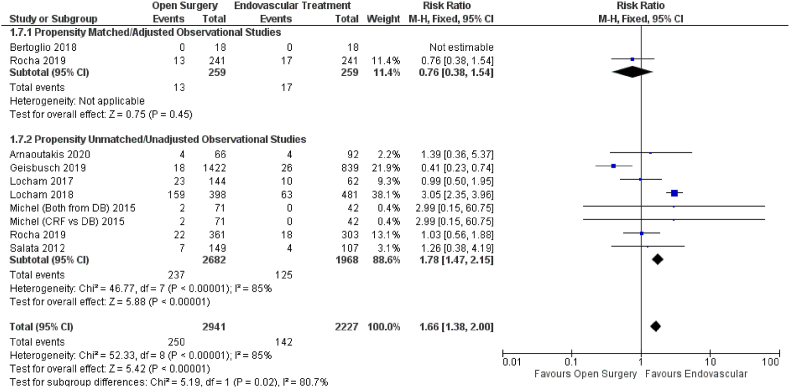
Forest plot comparing Open Surgery and Endovascular Treatment for Cardiac Complications.

The effect of open versus endovascular repair on the outcome of cardiac problems was studied in nine studies (Fig. 8). When compared to open repair, endovascular repair had a significant positive influence on cardiac complications (HR = 1.66; 95% CI: 1.38–2.00; P 0.00001). Subgroup analysis using propensity score unmatched (HR = 1.78; 95% CI: 1.47–2.15; P 0.00001) yielded similar results. However, the propensity matched data showed that open repair was preferred to endovascular repair (HR = 0.76; 95% CI: 0.38–1.54; P = 0.45).

## 4 Discussion

This meta-analysis collected data from twelve observational studies to compare outcomes of patients, such as stroke, all-cause mortality, renal failure, paraplegia, SCI, respiratory complications, in addition to cardiac complications, among those being operated on for endovascular or open repair of TAAA. Most outcomes favored an endovascular approach over open surgery, whereas, no outcomes were enhanced by the open repair technique. Similarly, for the subgroup analysis based on propensity-matched and unmatched data, most of the studies favored an endovascular approach over open repair. Interestingly, a contradicting finding was observed in the subgroup analysis of the propensity-matched data. It revealed that cardiac complications worsened when an endovascular technique was employed. One of the reasons for this unexpected observation could be the increasing imbalance, inefficiency, model dependence, and bias due to the propensity score matching. Thus, this finding is best ignored.

The group receiving endovascular repair mainly consisted of elderly individuals and those displaying a significant number of complications, such as chronic kidney disease and coronary artery disease. Due to the obvious absence of diverse end points according to statistics in the pooled analysis, selection bias has reduced the number of statistically different end points. Exclusively a randomized control trial could successfully identify whether open TAAA repair vs endovascular repair is better even though several observational studies sought to statistically resolve existing baseline imbalances.

The endovascular method, which was once only used for cases that could not be fully operated on, is now recommended for a broader group of people [10], strengthening the necessity for data comparing the aforementioned procedures. For individuals with connective tissue abnormalities and autopsies, open surgery remains the “validated” therapy. It is also crucial to consider the fragile structure of tissues in patients with connective tissue disorders during reconstruction of the aorta during surgery. TAAA dissections can now be handled endovascularly, owing to the recent introduction of endografts. This was evident in Bertoglio et al.'s [11] complete endovascular experience and Ferrer et al.'s [12] emergency operations using a prosthesis. Endovascular TAAA therapy would not have been a realistic option in this case in the earlier decades because the primary option was specially designed grafts, which took months to be produced. Whereas, with the recent advancements in the medical field, endovascular treatment has become a popular method of choice when compared to open surgery.

A study by Chuter et al. reported endovascular repair as relatively newer and less invasive treatment that has shown promising results in a wider patient cohort [13]. With limited exposure to intensive care unit, decreased length of hospital stay and lesser morbidity after surgery [14], endovascular treatment appears to be a safer alternative to open repair. Despite its popularity and potential benefits to open surgery extra care must be taken in patients diagnosed with connective tissue disorders. A previous study by Mohammadi. S et al. reported the development of hematoma with hemothorax and aortic dissection distal to the site of stent graft implantation. In addition to this an increased number of type 1 endo leaks was reported in study populations explored by Waterman et al. and 7. Marcheix B et al. and continued degeneration of the aneurysm even after stent implantation reported by M Norden et al. These findings raise the question of whether stent placement is a beneficial long-term solution to reduce the risk of further aneurysms in patients with connective tissue disorders. Hence it is recommended that a regular follow up should be taken for connective tissue disorder patients undergoing endovascular surgery.

The absence of uniformity among documentation of patient and disease characteristics, such as, history of dissection, aneurysm size and aneurysm extent is an important issue noted by this systemic review, preventing important insights about methodology outcomes, that can be a prospective source of bias [15]. Because TAAA repair morbidity and mortality varies greatly depending on the length of the aorta repaired, care should be exercised when comparing diverse groups with small sample populations [16,17]. In addition, as compared to surgical repair, endovascular treatment often necessitates a longer aortic coverage to repair identical aneurysm extent. Results of the comparisons across procedures discussed should be taken with hesitance due to unavailability of details regarding critical baseline features of inpatients, for example, the type and degree of aneurysm, existence of dissection, as well as the immediate need for surgery. As a result, clinical results should be reported according to the level of open surgical repair or endovascular aortic coverage, instead of the magnitude of condition of aneurysm for consistency.

The introduction of EVAR provides an alternate method for management of abdominal aortic aneurysm (AAA), with nearly 75% of AAAs receptive to it. EVAR's early benefits (reduced early death, limited stay at hospital) could be outweighed by longer-term morbidity (together with reintervention) and death, according to a number of studies but it is suggested as treatment in less fit individuals [4,14,[16], [17], [18]]. Due to the ambiguity of the situation, absence of uniformity and standardization among documentation of unfavorable events after operation was also discovered in this systematic research. No study recorded all of the predetermined outcomes being investigated (stroke, all-cause mortality, renal failure, paraplegia, spinal cord injury, respiratory complications, and cardiac complications), otherwise selected because of having a significant influence on the daily life of patients. We encourage the adoption of uniform criteria for recording SCI and other significant outcomes after open aortic repair of TAAA vs endovascular of TAAA since the severity of the neurological damage has an influence on prognosis [18].

The treatment of TAAA with endovascular therapy is rapidly evolving. Majority of reports will invariably contain instances from the researchers' early learning curve. As a result, we anticipated the decrease in occurrence of unfavorable events recently, which might influence our findings. Likewise, the open approach, has undergone a number of recent advancements [19,20]. As a result, both techniques will proceed to develop and change during upcoming years.

## 5 Strengths and limitations

The implementation of methodological approaches, such as a complete literature search, explicitly specified inclusion criteria, identical citation review, and data abstraction, is a strength of this work. The scarcity of comparable research in the literature is highlighted by our comprehensive study. A scarcity of relative data is present and there is no randomized control trial explicitly evaluating and comparing the techniques mentioned, despite the enormous quantity of case reports detailing outcomes of open TAAA surgery and, less extensively, endovascular TAAA repair. Furthermore, the majority of observational studies show disparities in baseline patient characteristics among groups (elderly individuals demonstrating greater comorbidities in endovascular cases) as well as study design variability. Provided that the sole observational research that propensity matched for baseline imbalances had more similar results, the possible better results following endovascular repair in the unmatched studies might be linked with additional unquantified prior inequalities favoring endovascular patients. This emphasizes the possibility for bias when depending upon data from observational studies. Finally, the considerably significant quantity of population-based studies included in this analysis that used administrative instead of comprehensive clinical data can possibly restrict solid conclusions regarding the outcomes stated.

## 6 Conclusion

A limited number of studies compare open vs. endovascular TAAA repair. Taking into consideration a small quantity of trials possessing a significant risk of bias, individuals getting endovascular therapy for TAAA may have better short-term results. These insights emphasize the necessity for greater and reliable evidences with reporting standards.

## Ethical approval

NA.

## Sources of funding

NA.

## Author statement

Aayat Ellahi conceived the idea and designed the study.

Fahd Niaz Shaikh, Haider Kashif, and Hamna Khan collected the data and analysed it.

Eman Ali, Bushra Nasim and Mariam Adil drafted the manuscript.

Zunera Huda conducted literature search and created the illustrations.

Muhammad Sameer Arshad revised the manuscript critically.

## Registration of research studies

Name of the registry: National Institute for Health Research (NIHR) International prospective register of systematic reviews (PROSPERO).

Unique Identifying number or registration ID: CRD42022341490.

Hyperlink to your specific registration (must be publicly accessible and will be checked): https://www.crd.york.ac.uk/PROSPERO/display_record.php?RecordID=341490.

## Consent

NA.

## Guarantor

Aayat Ellahi.

Jinnah Sindh Medical University.

## Provenance and peer review

Not commissioned, externally peer reviewed.

## Declaration of competing interest

None to declare.
