# Supplementary material for: Effectiveness of endovascular repair versus open surgery for the treatment of thoracoabdominal aneurysm: A systematic review and meta analysis
Source: Ann Med Surg (Lond). 2022 Sep 3;81:104477. doi: 10.1016/j.amsu.2022.104477 (PMC9486727; doi:10.1016/j.amsu.2022.104477)
Supplement: Multimedia component 3 [file mmc3.docx]

**SUPPLEMENTARY APPENDIX**

**Supplemental Table S1: Quality Assessment of included studies using Newcastle Ottawa Scale**

| **Study/Score** | **Selection** | | | | **Comparability** | | **Outcome** | | |  | |
| --- | --- | --- | --- | --- | --- | --- | --- | --- | --- | --- | --- |
|  | **S1** | **S2** | **S3** | **S4** | **C** |  | **O1** | **O2** | **O3** | **Total** |  |
| Ferrer 2016 | * | * | * | * | * |  | * | * | * | 8 |  |
| Bertoglio 2017 | * | * | * | * | * |  | * | * |  | 7 |  |
| Greenberg 2018 | * |  | * | * | * |  | * | * | * | 7 |  |
| Michel 2015 | * | * | * |  | * |  | * |  |  | 5 |  |
| Salata 2012 | * | * |  | * | * |  | * | * |  | 6 |  |
| Sachs 2010 | * | * | * | * | * | * | * | * |  | 8 |  |
| Locham 2017 | * | * | * | * | * |  | * | * |  | 7 |  |
| Locham 2018 | * | * | * |  | * |  | * | * | * | 7 |  |
| Rocha 2021 | * | * | * | * |  | * | * | * | * | 8 |  |
| Arnaoutakis 2020 | * | * |  | * | * |  | * |  |  | 5 |  |
| Geisbusch 2019 | * |  | * |  | * |  |  | * | * | 6 |  |
| Kang 2019 | * |  | * | * |  |  | * |  | * | 5 |  |
